# Endocrine disrupting chemicals and male fertility: from physiological to molecular effects

**DOI:** 10.3389/fpubh.2023.1232646

**Published:** 2023-10-10

**Authors:** Marwa Lahimer, Maria Abou Diwan, Debbie Montjean, Rosalie Cabry, Véronique Bach, Mounir Ajina, Habib Ben Ali, Moncef Benkhalifa, Hafida Khorsi-Cauet

**Affiliations:** ^1^ART and Reproductive Biology Laboratory, University Hospital and School of Medicine, CHU Sud, Amiens, France; ^2^PERITOX-(UMR-I 01), UPJV/INERIS, UPJV, CURS, Chemin du Thil, Amiens, France; ^3^Exercise Physiology and Physiopathology: from Integrated to Molecular “Biology, Medicine and Health” (Code: LR19ES09), Sousse, Tunisia; ^4^Fertilys, Centres de Fertilité, Laval and Brossard, QC, Canada; ^5^Service of Reproductive Medicine, University Hospital Farhat Hached, Sousse, Tunisia; ^6^Laboratory Histology Embryology, Faculty of Medicine Sousse, University of Sousse, Sousse, Tunisia

**Keywords:** endocrine disrupting chemicals, pesticides, spermatogenesis, sperm characteristics, hormonal disorders, epigenetics modification

## Abstract

The deleterious effects of chemical or non-chemical endocrine disruptors (EDs) on male fertility potential is well documented but still not fully elucidated. For example, the detection of industrial chemicals’ metabolites in seminal plasma and follicular fluid can affect efficiency of the gametogenesis, the maturation and competency of gametes and has guided scientists to hypothesize that endocrine disrupting chemicals (EDCs) may disrupt hormonal homoeostasis by leading to a wide range of hormonal control impairments. The effects of EDCs exposure on reproductive health are highly dependent on factors including the type of EDCs, the duration of exposure, individual susceptibility, and the presence of other co-factors. Research and scientists continue to study these complex interactions. The aim of this review is to summarize the literature to better understand the potential reproductive health risks of EDCs in France.

## 1. Introduction

Infertility is defined as a failure to fulfil a successful pregnancy after 12 months from unprotected intercourse. Nearly 17 to 20% of couples have a difficulty to conceive naturally around the world (1). Based on the epidemiological study published in 2015, the World Health Organization estimates that nearly 190 million people suffer from infertility worldwide (2). More than half of cases were attributed to male factor (3).

Endocrine disrupting chemicals (EDCs) exposure may affect male fertility at multiple levels, from sperm production and quality to the morphology and histology of the male reproductive system. It has been suggested that exposure to EDCs may result in impaired sperm motility, concentration, volume, and morphology, as well as increased sperm DNA damage (4).

The literature reported that male fertility decline can be associated with the occupational and the environmental factor (5, 6). The Human body is directly or indirectly exposed to a variety of toxins having the potential to disrupt endocrine homeostasis. The negative effects of EDCs on spermatogenesis and male fertility, have been investigated and demonstrated in clinical experiments and epidemiological studies. In fact, EDCs alter sperm function by targeting testicular development. In addition, they have an impact on the hypothalamic–pituitary axis by affecting estrogen and androgen receptors, production of reactive oxygen species (ROS), may induce epigenetic modifications or direct effect on spermatozoa and other cells of testicular tissue (7). A wide range of pesticides have been identified as EDCs. Many studies investigated effects of pesticides exposure on sperm parameters decline and DNA integrity defects (5, 6, 8, 9). However, several studies reported a reduction of some sperm parameters such as concentration, typical form and others conversely showed no impact on sperm parameter and neither DNA integrity (10, 11). The objective of the present review is to summarize the current knowledge about the impact of EDCs on male fertility, including the current situation in France.

## 2. The hypothalamo-pituitary-gonadal axis

It is well known that the hypothalamo-pituitary-gonadal axis is a hormone-regulating structure containing the hypothalamus, pituitary gland, and gonadal glands, crucial for reproduction (12). Regulation of the reproductive axis begins at the level of the hypothalamus.

The hypothalamus produces and releases gonadotropin-releasing hormone (GnRH), which stimulates the pituitary gland to produce and release follicle-stimulating hormone (FSH) and luteinizing hormone (LH). These hormones then stimulate the testis which in return produces sex hormones such as testosterone (13, 14). The testis has two main functions. Firstly, it produces spermatozoa and secondly it has an endocrine function that is essential for the development and function of male reproductive organs, as well as secondary sexual characteristics (15).

The endocrine system plays a crucial role in the regulation of the reproductive function. Any alteration of the functionality can lead to an imbalance and alteration of male reproductive health (16). The hypothalamic–Pituitary-Gonadal axis is the target of EDCs.

## 3. Endocrine disrupting chemicals: definition

The ANSES (Agence nationale de sécurité sanitaire de l’alimentation, de l’environnement et du travail) and EPA (Environmental Protection Agency) both define an endocrine disruptor (ED) as a chemical substance that can interfere with the functioning of the endocrine system of humans or animals and cause adverse health effects in an organism or its progeny. These effects can occur at very low doses and may include developmental, reproductive, neurological, and immune-related effects. The ANSES and EPA both have lists of known or suspected EDs. Any chemical that exhibits similar effects on the endocrine system will be considered a potential ED and subject to further evaluation and regulation (17, 18).

Colborn et al. (19) defined the term “endocrine disruptor” and issued a consensus statement (known as Wingspread Statement) about the Impacts of Endocrine Disrupting Chemicals (EDCs) on environmental and human health (20). Over recent years, many environmental EDCs have been shown to disrupt the actions of hormones (synthesis, secretion, transport, and binding) or eliminate their effect. Eventually, it was discovered that the most common mechanism of action of EDCs is to imitate endogenous hormones and compete with their Nuclear Receptors (NRs) as agonists or antagonists. Some of these compounds are naturally occurring (such as phytoestrogens), but most are synthetic chemicals that are released into the environment through human activity without prior knowledge of their impact on ecosystems or human health (20). There are many chemicals and pesticides commonly used that have been classified as EDs by the ANSES and EPA such as polychlorinated bisphenyls (PCB), bisphenol A (BPA), phthalates and their metabolites (dibutyl phthalates (DBP) and Di-(2-ethylhexyl) phthalate (DEHP)), alkyl phenols, Glyphosate, dichlorodiphenyltrichloroethane DDT and Methoxychlor (Table 1). Phthalates are chemical substances present in everyday objects in the French general population’s daily environment (e.g., cosmetics, varnishes, paints, solvents, textiles, stove adhesive coatings, plastic toys) (29). It is known that the effects of these chemicals on the endocrine system may vary depending on the dose, route of exposure, and the individual’s susceptibility (17, 18, 30). Environmental endocrine disrupting chemicals (EDCs) can interfere with the biosynthesis and actions of androgens, which are male sex hormones that play a critical role in the development and maintenance of the male reproductive system (31). EDCs can disrupt the cellular processes that control the production of androgens in Leydig cells, including the transport and delivery of cholesterol into mitochondria, the expression or activity of steroidogenic enzymes, and the binding of androgens to the androgen receptor (AR). This disruption can result in incomplete masculinization and malformations in the male reproductive tract of both humans and animals. The effects of EDCs on androgen biosynthesis and actions can vary depending on the specific chemical and the timing and duration of exposure. In some cases, the effects may be reversible if exposure to the EDC is stopped, but in other cases, the effects may be permanent (32–34).

**Table 1:** Impact of some EDCs on animals’ reproductive system function.

References	EDCs	Population	Impact on animals’ reproductive system
Chen et al. (21)	Chlorpyrifos	mouse-derived spermatogonial cell lines (GC-1), Sertoli cell lines (TM4) and Leydig cell lines (TM3) of mice	Apoptosis, increased reactive-oxygen-species (ROS) production and lipid peroxidation (MDA), reduced mitochondrial-membrane potential and increase in phosphorylated-AMP-activated-protein-kinase (p-AMPK) levels.
Li et al. (22)	Chlorpyrifos	Rats	Modification of sperm, serum hormones, oxidative stress in the testis, and enzyme activity related to spermatogenesis, decreased total sperm count, serum testosterone and gonadotropin levels.
Dobrzyńska (23)	BPA	Mice	Decreased testosterone levels
Tiwari and Vanage (24)	BPA	Rats	- Impaired sperm motility, increased sperm DNA damage - Decreased sperm counts
El-Beshbishy (25)	BPA	Rats	Inhibition of sperm motility and motion kinematics by significantly decreasing ATP levels in spermatozoa.
Rahman et al. (26)	BPA	Rats	Disrupts spermatogenesis.
Lassen et al. (27)	BPA	Rats	Binds to and acts as an antagonist of the androgen receptor.
Harper et al. (28)	Atrasine	Mice	- Decreased epididymal sperm concentration - Affects both metabolic and reproductive characteristics

## 4. Endocrine disrupting chemicals in France

A wide range of EDCs is used in agriculture in France, as pesticides. Seven years ago, 68 thousand tons of pesticides were purchased, making France the third largest user of pesticides in the world and the largest user in Europe (35). Several studies assessed the genotoxicity of EDCs on human body health (36–41). EDCs are neurotoxic with adverse effects on brain maturation. As an example, in our previous review, we detailed the impact of the pesticide Chlorpyrifos (CPF) on the different components of the gut-microbiota–blood–brain barrier axis while the first identified effect of CPF was its neurotoxicity killing pests: the inhibition of acetylcholinesterase (42, 43). An experimental study conducted in 2018 by Laporte and collaborators included 21 female (10 weeks old) and 10 male of Wistar rats that received daily CPF exposure (1 mg/kg, *per os*) during gestation and lactation (44). The maternal exposure to CPF had a negative impact on intestinal homeostasis of 51 male rats of the offspring (Wistar rats). Therefore, despite the lack of direct contact with pesticides except through breast milk until weaning, maternal exposure to the pesticide appeared to delay fetal and postnatal weight gain (45, 46). In addition to all of these effects, CPF is currently considered an EDC (47). It was shown that EDCs impact on human health can go beyond dysregulation of the endocrine system and affect physiological barriers by targeting the gut microbiota (42).

A retrospective study included 633 couples undergoing IVF cycles, who live in Picardy (northern France) and are highly exposed to pesticides. The authors reported an association between the pesticide’s exposure levels, the embryological and clinical outcomes that were also analyzed for both groups. Results showed a decrease in, embryo cleavage, ongoing pregnancy, and live birth rate and an increase in early miscarriage (48).

A study of 51 male rat pups reported the effect of maternal exposure to CPF or a high fat diet (HFD) (in addition of breast milk, progeny was fed a HFD). The indirect exposure to CPF was associated with changes in the offspring metabolic activity in the liver and reduction in the expression of genes coding for enzymes involved in lipid or glucose metabolism (49).

Once a pesticide or its metabolites circulate in the human body, it may create a functional disorder such as cardiotoxicity. Then, the cardiovascular toxicity may lead to morphological, biological, physiological, histopathological, and serological changes (50).

Based on available epidemiological data published in literature, exposure to pentachlorophenol (PCP) was associated with an increased risk of non-Hodgkin lymphoma, which is also reported in a cohort of US pesticide manufacturing workers exposed to PCP (51). A review of articles published between 1982 and 2021 suggests the involvement of heavy metals lead, cadmium, and copper in male and female infertility. It can result in a decline in semen quality and a reduction in the number of oocytes reaching metaphase II (52). Another study that analyzed 144 articles showed that environmental and occupational exposure is associated with a decrease in conventional sperm parameters, sperm DNA damage and chromatin defect (53).

The European Food Safety Authority (EFSA) re-evaluated bisphenol A (BPA) and subsequently reduced the tolerable daily intake (TDI) from 4 micrograms per kilogram of body weight (μg/kg bw) per day to 0.2 nanograms (ng) per kilogram of body weight per day (54).

In France, Sirot et al. (55) reported an assessment of contamination levels in infant food and the resulting exposures for children under 3 years old. They reported that certain children were found to have exposure levels to BPA that surpassed the minimum toxicological value set by the French Agency for Food. The Environmental and Occupational Health & Safety has established a minimum toxicological value of 0.083 μg/kg bw per day. However, the temporary TDI set by the EFSA at 4 μg/kg bw per day was never surpassed.

## 5. Physiological effects of EDCs on male infertility

### 5.1. Hypothalamic–pituitary axis

Pesticides, as endocrine disrupting chemicals, can also alter the reproduction function affecting hypothalamic and pituitary axis which may alter the secretion of GnRH, LH, and FSH widely by modifying the feedback of endogenous hormones. Several studies have demonstrated that low-dose of DDT and methoxychlor can result in a reduction of hypothalamic and pituitary functions (56, 57).

Several experimental studies on rats have reported that perinatal, juvenile, or adult exposure to EDCs, disrupt the hypothalamic control of pituitary gonadotropin synthesis leading to a disruption of gonadal steroid production and androgens cyclicity (58–62). These studies suggest that EDCs exposure affects the expression of GnRH and kisspeptin, influences the pulsatile launch of GnRH, and interferes with the regulation of gonadotropin production from the pituitary axis (63). These impacts can arise from periodic exposures during perinatal, juvenile increase, and adulthood at low concentrations and lead to disruption of steroidogenesis, estrous cyclicity and the onset of puberty (64).

Hyperthyroidism is characterized by increased total thyroxine (T4) levels which lead to an increase in sex hormone binding globulin (SHBG) in circulation and a decrease in the level of testosterone (65).

These environmental substances can disrupt exclusive tiers of the hypothalamic–pituitary axis, including the synthesis, metabolism, and organic effects of thyroid hormones. Given the significance of this gland for homeostasis, it is far crucial to better understand the mechanisms involved in the action of EDCs on thyroid function (66).

Several studies have shown that phthalates reduce testosterone production and testicular expression of genes and proteins involved in steroid synthesis, but few studies have described alterations in the expression of PPARs (peroxisome proliferator-activated receptors) in testes and other tissues. Phthalates and their metabolites have been extensively studied for their impact on sexual development and fertility because of their hormone-like effects (67). Several studies, focusing on the biochemical (enzymes implication) effects, have suggested their involvement in PPARs pathways, to have a better understanding of these physiological modifications. These compounds are known to activate these receptors, specifically PPARα and PPARγ, which are nuclear receptors involved in regulating various physiological processes, including lipid metabolism and inflammation (68, 69). By acting as agonists for PPARα and PPARγ, thus disrupting their normal function, these chemicals can lead to dysregulation of the hormonal balance and perturbations in endocrine signaling pathways (70). Consequently, this disruption can potentially affect the expression of aromatase (encoded by CYP19 gene), an enzyme crucial for estrogen synthesis, through conversion of androgens to estrogens in gonadal and extra-gonadal tissues. This enzyme can convert testosterone to estradiol (71). Dysregulation of aromatase can disturb the balance of sex hormones, contributing to reproductive issues and infertility. In fact, both deficiency and excess of aromatase may result in many disorders related to sex hormones profile (72). These findings highlight the complex mechanisms through which phthalates can impact sexual development and fertility, with PPARα and PPARγ pathways playing a pivotal role in these adverse effects.

In the context of male infertility, certain obesogenic EDCs like Bisphenol A (BPA) (73), Phthalates (74), Perfluoroalkyl Substances (PFAS) (75), Polychlorinated Biphenyls (PCBs) (76), Polybrominated diphenyl ethers (PBDEs) (77), and Atrazine (28) can impact male infertility and might be linked to reduced sperm quality, hormonal imbalances, and metabolic disruptions that contribute to obesity (78).

Numerous studies have been carried out in animal models to explore the impacts of various factors on physiological processes (Table 1). There are a few observational studies conducted in humans (Table 2). Animal studies provide valuable insights to better understand the implications of these factors in human health (89, 90).

**Table 2:** Impact of some EDCs on human reproductive system function.

References	EDCs	Population	Impact on human reproductive system
Perez et al. (79)	Diethylstilbestrol	1,085 DES-exposed and 1,047 unexposed men	Affects the likelihood of never fathering a pregnancy or live birth
Palmer et al. (80)	Diethylstilbestrol	3,067 men (1,638 exposed, 1,429 unexposed)	Varicocele, structural abnormalities of the penis, urethral stenosis, benign prostatic hypertrophy, or inflammation/infection of the prostate, urethra, or epididymis.
Zhou et al. (81)	bisphenol A	308 young men	- Disrupts spermatogenesis. - Binds to and acts as an antagonist of the androgen receptor.
Bloom et al. (82)	Phthalate	473 men	Lower total sperm counts and concentrations, lower sperm motility.
Phillips (74)	Phthalate	men	- Low testosterone levels in men. - Affects testicular function - Metabolic abnormalities in men: abdominal obesity and insulin resistance
Meeker et al. (83)	Parabens	132 men	Sperm DNA damage.
Chen et al. (84)	Phenols	877 idiopathic infertile men and 713 fertile controls	Abnormal semen parameters
Dalvie et al. (85)	DDT	60 workers	Abnormal testes disposition, negatively associated with sperm count
Lahimer et al. (86)	Pesticides exposures	671 men	Vitality and motility decline, Sperm DNA damage (increased fragmentation index)
Chevalier et al. (87)	BPA	52 cryptorchid (26 transient, 26 persistent) and 128 control boys	- Deleterious impact on fetal testicular descent during specific windows - Disturbance of INSL3 levels
Shen et al. (88)	fine particulate matter	2,332 Participants	Shortened AGD in newborns
Vuong et al. (77)	polybrominated diphenyl ethers (PBDEs)	206 children	Obesogenic effect, increased adiposity measures.

### 5.2. Testicular cells: Leydig and Sertoli cells

Several animal experimental studies working on xenobiotics inducing a testicular disorder, reported that only some have caused reproductive damage in men. Pham and colleagues in 2019 exposed the pregnant mice and their offspring to different doses of glyphosate and a specific glyphosate based on herbicide (GBH). The doses tested were 0.5 mg/kg/day, 5 mg/kg/day and 50 mg/kg/day. The exposure began at embryonic day 10.5 (E10.5) and continued until 20 days postpartum. The male offspring of the treated mice were analyzed at different ages: 5 days old, 20 days old, 35 days old, and 8 months old. The study outcome showed that glyphosate exposure (but not GBH exposure) led to changes in the structure of the testes (testes morphology) in males that were 20 days old and resulted in decreased serum testosterone concentrations in males that were 35 days old. Additionally, they found that the spermatozoa number decreased by 89 and 84% in 0.5 and 5 mg/kg/day of GBH and glyphosate groups, respectively. This data suggests that, at low doses, glyphosate and GBH have endocrine disrupting effects on male reproduction (91).

Dysregulation of estrogen synthesis or estrogen receptor function in the efferent ducts results in testicular inflation and seminiferous tubular atrophy. Subsequently, testicular atrophy is provoked after chronic or sub chronic exposures to environmental toxicants, lesions in efferent ducts and head of the epididymis which can lead to permanent infertility (92).

Numerous studies showed that pesticide exposure damaged some testis functions (secretion of androgens in Leydig cells or Inhibin B in Sertoli cells). A study published in 2012 showed that Leydig cells are damaged after 1 to 48 h of exposure to glyphosate-based herbicide. Within 24-48 h, this toxic also damaged the other cells, mainly by necrosis, conversely to glyphosate alone which exerted a toxic effect on Sertoli cells. At long term exposition, it also induces apoptosis in germ cells and in Sertoli cells. At lower nontoxic concentrations of glyphosate (1 ppm), the main impact is a testosterone decrease by 35%. These results show that the pesticide has an endocrine impact at very low environmental doses (93). In addition to pesticides, scientific studies have shed light on how phthalates disrupt testosterone production in Leydig cells in the testes, impacting fetal sexual development. Detrimental effects of phthalate exposure on Steroidogenic Acute Regulatory (StAR) protein expression have been highlighted. In fact, phthalates interfere with Leydig cells by decreasing the production of StAR protein. This reduction in StAR protein levels hinders the transport of cholesterol into the mitochondria, a critical step in the synthesis of testosterone (94). This disruption in testosterone production during fetal development can have profound consequences on sexual differentiation and maturation, potentially leading to reproductive health issues later in life. The role of phthalates in fetal sexual development is a complex area of research, and recent studies continue to underscore the significance of understanding the mechanisms through which these chemicals impact endocrine function and reproductive outcomes.

A growing body of evidence suggests a correlation between exposure to Bisphenol A (BPA) and the progression of prostate cancer. The potential impact of BPA on the advancement of this malignancy raises concerns about its role in influencing disease development and severity (95). A Spanish study included 4,812 participants (547 breast cancer cases, 575 prostate cancer cases and 3,690 sub-cohort participants) and aimed to analyze serum BPA concentrations. The study outcome reported that elevated level of serum BPA is associated with 40% increase in risk of prostate cancer while no significant association have been found between BPA and breast cancer (96).

It’s well known that BPA exposure is associated with reproductive health disorders including cryptorchidism, a condition characterized by the incomplete descent of the testes into the scrotum (97). A prospective study was conducted on 98 children with congenital unilateral cryptorchidism and 19 healthy boys. The study findings suggest a significant increase in BPA level in the cryptorchid group 61.4% (98). Furthermore, Shi et al. reported that prenatal exposure of male mice to BPA leads to a decrease in sperm quality including sperm count and sperm motility. Additionally, BPA has been linked to a disturbance in the distribution of spermatogenesis stages (99). Regarding the pathogenesis impact of BPA, research has suggested that BPA has a positive association between pesticide levels and risk of hypospadias. A study including 668 mother–son pairs from Southern Spain indicated a significant negative impact between exposure to BPA and other phenols during pregnancy and male genital development including cryptorchidism and hypospadias (100, 101).

According to its estrogenic properties, BPA exposure during critical windows of fetal development could potentially disrupt the normal hormonal signaling pathways such as INSL3 (insulin-like 3) (87). This hormone is involved in the development of the male reproductive system especially in the release of testosterone and the descent of the testes into the scrotum before birth (102). A French case–control study has demonstrated the negative correlation between blood BPA levels and INSL3 levels leading to cryptorchidism. This study included a total of 52 cryptorchid (26 transient, 26 persistent) and 128 control boys from southern France. The study outcome revealed that chronic fetal exposure to BPA leads to the disturbance of INSL3 pathway (INSL3 reduction levels, *p* = 0.01; *R*^2^ = 0.05) and the occurrence of cryptorchidism (87). Another experimental study published by Desalegn confirmed that perinatal exposure to EDCs is associated with increased congenital cryptorchidism (103). Anogenital distance (AGD) including anoscrotal distance (ASD) and anopenile distance (APD) in males can also be link to perinatal exposure to EDCs (88).

**Figure 1:**
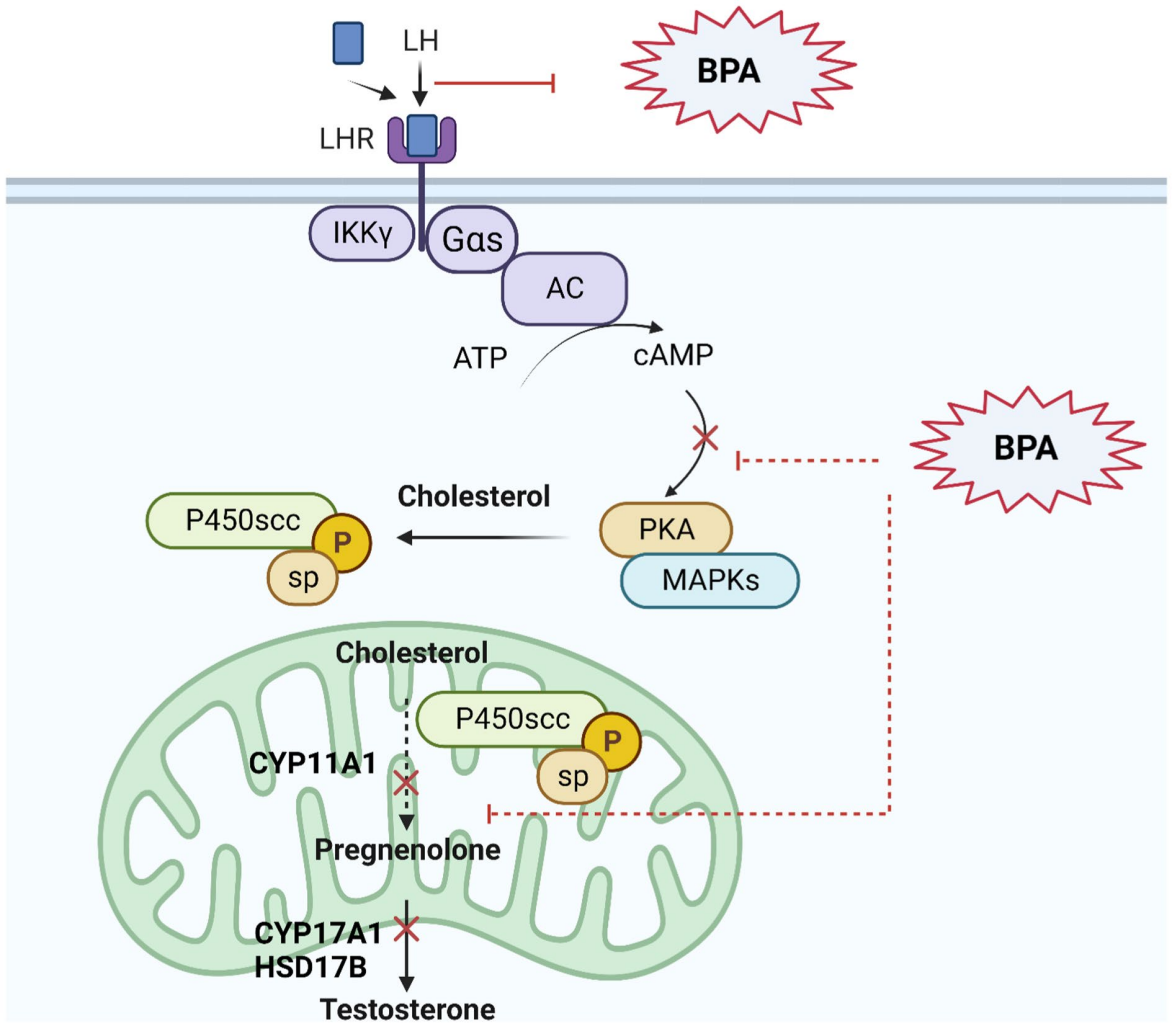
BPA’s effect on androgen signaling pathway in Leydig cells. BPA as an endocrine disruptor has an androgenic property to interact with androgen receptors in cells, with lower affinity than natural androgens. BPA can bind to androgen receptors and militate or inhibit certain androgenic responses, potentially leading to disruptions in hormone signaling including testosterone synthesis as described in figure: Upon binding to LH, the LH receptor undergoes a conformational change that activates a G protein, usually a Gαs protein. The activated G protein (Gαs) increases cAMP levels and activates PKA, which then phosphorylates various proteins within the Leydig cell. This includes the cholesterol side-chain cleavage enzyme (P450scc), resulting in the conversion of cholesterol to pregnenolone. Pregnenolone is then converted through a series of enzymatic reactions to produce testosterone.

Evidence suggests that BPA could potentially impact estrogen and androgen receptors, and interfere with LH binding and testosterone synthesis in Leydig cells (104) (Figure 1).

**Figure 2:**
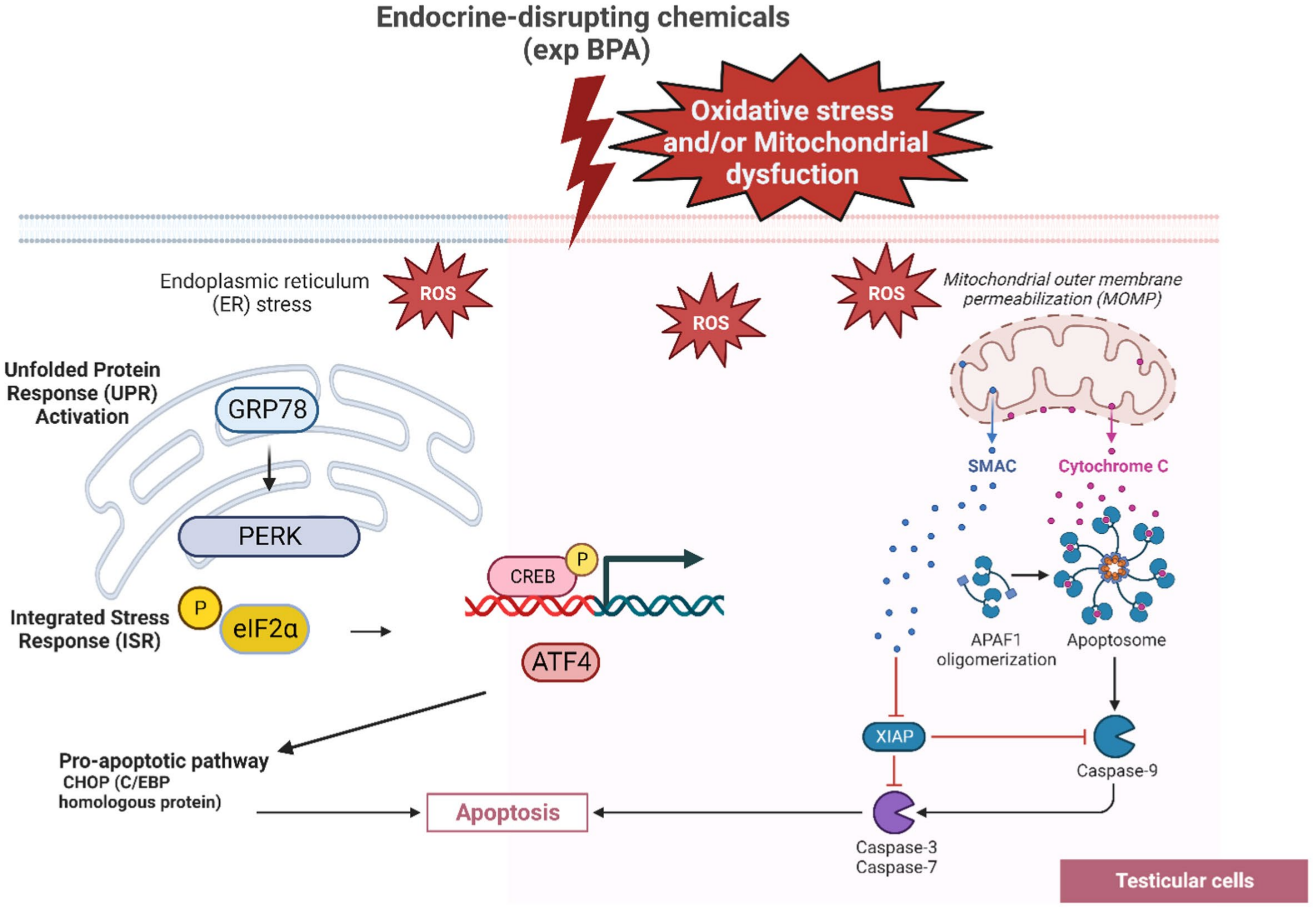
The GRP78-PERK pathway leading to testicular cell apoptosis as a response of BPA’s oxidative stress. The interaction between BPA exposure, ROS generation, UPR activation, stress transducers and apoptosis in testicular cells highlights the complicated cellular responses to environmental toxins and endocrine disruptors. As a response to ER stress caused by ROS, the chaperone protein GRP78 is recruited to bind to unfolded/misfolded proteins allowing PERK to become activated. PERK activation leads to the phosphorylation of eIF2α (eukaryotic translation initiation factor 2α). The phosphorylation of eIF2α leads to a global reduction in protein synthesis ATF4 transcriptionally upregulates genes involved in apoptotic pathways, including CHOP (C/EBP homologous protein, also known as GADD153).

Endoplasmic reticulum (ER) stress has been shown to contribute in defective spermatogenesis in both animal models and humans (105). Several studies have reported a significant increase in ROS in infertile men compared to fertile men (105, 106). Exposure to environmental toxins like BPA can induce ER stress and contribute to testicular cell damage leading to impaired spermatogenesis and apoptosis (107). Elevated level of ROS can trigger protein folding, Unfolded Protein Response (UPR) activation and affect stress transducers like GRP78, PERK and potential mitochondrial dysfunction (106). The intrinsic pathways of apoptosis including mitochondrial pathway and ER stress pathway within testicular cells were described in Figure 2.

### 5.3. Spermatogenesis

Nowadays several epidemiological and clinical tests reported that semen quality represents a clear decline over the year. Animal studies are widely used and are often the first sight of the impact of EDCs on reproductive or developmental effects in humans. Xenobiotics connotatively alter human reproduction by affecting the development of gonads and gametogenesis. In particular, spermatogenesis is the process by which sperm cells are produced in the testes, and EDC exposure can potentially disrupt this process, leading to spermatogenesis failure or impaired sperm production (108). The spermatogenic cycle takes 74 days in humans (35 days in mice and 52 days in rats). This process may be affected by chemicals disruptors at any stage of cell genesis, giving rise to declined sperm parameters: counts, morphologically abnormal sperm, impaired stability of sperm chromatin or sperm DNA damage (109). This study shows that the number of spermatogonia affected will increase with repeated exposure to xenobiotics, which can only be detected by histopathology. Subsequently, all other spermatogenic cells are undifferentiated, altered and then depleted, which may result in the decrease of testis weight and sperm count (109). The mechanisms underlying these disruptions are complex and can involve several pathways including hormone disruption (110), Sertoli cells dysfunction, Leydig cells dysfunction (111), epigenetic changes (112), oxidative stress (113, 114), and disruption of blood-testis barrier (115, 116).

The main effects of EDCs on spermatogenesis and male fertility that have been demonstrated are the structural damage of the testis (vasculature and blood-testis barrier) and the harm of Leydig and Sertoli cells (4). Bahri et al. (117) found a significant reduction in the semen quality of North African men in a retrospective study of 20,958 sperm analyses (117).

Semen volume (mL), percentage of motile sperms, sperm concentration (millions/mL) and percentage of sperms with normal morphology are by far the major questionable factors for male fecundity. Most occupational semen studies compared semen quality between exposed and unexposed workers. The exposed participation has 40–60% lower semen quality than controls. This may introduce the effect of EDCs in fertility problems (118). A meta-analysis from 64 papers (8) showed a negative association between pesticides exposure as EDCs and sperm characteristics. Therefore, EDCs especially organochlorines and organophosphates affect frequently some sperm parameters like total sperm count, sperm motility, and sperm morphology (8). The investigation of 2,122 patients reported an association between altered sperm parameter and occupational pesticides exposure. They found that semen volume, motility, and vitality were decreased in men exposed to pesticides (119). However, they suggested that some pesticides could affect post testicular sperm maturation, in which epididymis, prostate, and seminal vesicles are particularly involved (119). On the contrary, our study analyzed a total of 671 men living in a region (Picardy region of France) highly exposed to pesticides and revealed no significant impact of pesticides exposure on some conventional sperm parameters (86). Delgouffe and colleagues highlighted important findings in their study published in 2023. The investigation regarding the impact of gonadotoxic treatment on testicular tissue, spermatogenesis, and reproductive hormone disruption. The outcome reported a reduced testicular volume in 9 among 12 treated patients, that can indicate testicular damage or dysfunction. Additionally, a total of 10 patients showed some degree of reproductive hormone disruption. This disruption can explain the disrupting impact of the gonadotoxic treatment on the endocrine system. In contrast, one of the most remarkable findings of this study is that, despite the gonadotoxic treatment and the potential adverse effects on the testes, ongoing spermatogenesis was observed in 8 out of the 12 patients (120).

Interestingly, primary testicular failure known as primary hypogonadism, occurs when the testes are unable to produce enough testosterone and other hormones despite normal or elevated levels of gonadotropins like FSH (serum FSH levels ≥10 IU/L). Based on this concept, the study of Kanbar et al. (121) revealed a higher incidence of primary testicular failure after gonadotoxic treatment (121).

Plastic Derived EDCS are common in domestic use. DHEP and BPA are currently found in urine of men and they negatively impact sperm quality (sperm count, morphology and motility) (82). A systematic review and meta-analysis of 7,825 individuals and 479 individuals, respectively, performed the impact of up to 12 families of EDCs on male infertility (semen quality, DNA integrity and pregnancy rate or fecundability), the data analysis showed a high heterogeneity among studies. The metanalysis revealed a positive association between polychlorinated biphenyls (PCB153) exposure and sperm count, but no association between bisphenol A, PCB153 and the rest of sperm parameters analyzed (122). After the Elfe study conducted in 2011 to measure the exposure levels of bisphenol and phthalates in women who gave birth in France (123, 124). The Esteban study aimed to estimate the bisphenols and phthalates exposure of children (*n* = 1,104, 6–17 years-old) and adults (*n* = 2,503, 18–74 years-old) of the French population between 2014 and 2016 (29). This study was based on the quantification of these compounds and their metabolites in urine [phthalates metabolites are almost completely excreted via urine (125, 126)], mainly by liquid and gas chromatography followed by mass spectrometry. Results were represented as reference values of exposure (RVs) that reflect the concentration of the chemical substance the population is exposed to. Results showed higher RVs for phthalates than bisphenols in children and adults. Phthalates metabolites with the highest RVs were the mono-isobutyl phthalate (MiBP) and the mono-isobutyl phthalate (MEP) (129 and 402 μg/L, respectively, in adults’ urine; 162 and 492 μg/L, respectively, in children’s urine). Exposure to phthalates was also evaluated in tap and raw water across 101 French departments (from November 2015 to July 2016). Dibutyl phthalate (DBP) and diethyl phthalate (DEP) were, respectively, the most frequently detected pollutants in tap water and raw water at a maximum concentration of 1,300 ng/L and 255 to 406 ng/L (less than ten times the limits quantification LOQ) (127).

*In vitro* exposures of sperm for 90 min to phthalate at different concentrations (1, 10, 100, and 500 μg/mL) showed that phthalate significantly reduces the sperm motility and hyperactivity by preventing the sperm ability to generate ATP. Also, short-term phthalate exposure (>10 μg/mL) generate abnormal capacitation and the acrosome reaction by increasing protein tyrosine phosphorylation via a protein kinase-A-dependent pathway. Otherwise, phthalate exposure (particularly 10 μg/mL) significantly affected fertilization and embryonic development. The findings showing by Amjad and his colleagues in 2021 indicate that the phthalate mixtures negatively affected sperm motility, capacitation and acrosome reaction, which provoked a poor fertilization rates and defect embryonic development (128). Plastic Derived EDCs are common in domestic use. DHEP and BPA are currently found in urine of men and they negatively impact sperm quality (sperm count, morphology and motility) (82).

While genetic factors certainly play a crucial role in semen quality decline, environmental factors, including exposure to EDCs like triclosan (found in antibacterial soaps, toothpaste, hand sanitizers, and even some cosmetics), have been hypothesized to disrupt the endocrine system and potentially affect the development and function of the male reproductive system (129). A prospective study in China including 443 couples reported that increased levels of triclosan in male urine have been linked to reduced fecundity (OR 0.77; 95% CI, 0.62–0.97). Furthermore, there was a notable rise in the risk of infertility, indicated by an odds ratio (OR) of 1.6 (95% CI, 1–2.6) (129).

## 6. Molecular effects of EDCs on male infertility

### 6.1. Sperm DNA integrity

In typical and healthy spermatozoa, the chromatin is presented as a sequence of nucleotides well compacted and devoid of DNA strand breaks or base mutation (130). During chromatin compaction (the processes that give a higher DNA stability and protect the double strand in its path through the male and female reproductive tracts) (131), the protamine replaces 85% of histones. Any abnormalities in protamine replacement, deficiency and mispackaging can provoke an error in DNA compaction and lead to error in spermiogenesis process which contributes to premature nuclear condensation and spermatozoa immaturity (132, 133).

The DNA fragmentation is a break at the single DNA strand defined as single-strand DNA breaks or at the double DNA strands and termed as double-strand DNA breaks, generating free 5′–3′ ends affecting DNA sequences (134).

As mentioned in literature, the causal factors of DNA fragmentation are diverse. They include pathology like varicocele, lifestyle, environmental and occupational exposure to pesticides. These factors increase ROS levels in cells which lead to an oxidative stress. The ROS attacks the nucleoproteins, provoke a lipid peroxidation and a damage in DNA (fragmentation and oxidation) (3). In addition, chromatin compaction defects make the spermatozoa more sensitive to the attack of ROS (135). Many EDCs have been found to affect male fertility at multiple levels, including sperm production, quality, and morphology, as well as the structure and function of the male reproductive system (82). Exposure to EDCs has been associated with a decline in sperm quality and quantity, as well as an increase in DNA damage in sperm (136). Preclinical studies with male rats showed that exposure to BPA increase the lethal mutation rate during fourth and sixth week of mating intervals at 5.0 mg/kg body weight dose of BPA. The results indicate this latter impacts the germ cell and damages the sperm DNA (24). A study assessed the impact of phthalates on 379 by evaluating the men urinary concentrations of phthalate metabolites and the sperm DNA damage. The results showed that sperm DNA damage was associated with diethyl phthalate’s metabolites [Monoethyl phthalate and Mono-(2-ethylhexyl) phthalate] (137). In addition, in 2011, Meeker and collaborators demonstrated the association between the urinary concentrations of parabens, serum hormone levels, semen quality parameters, and sperm DNA damage, reporting that there is no significant association between the urinary parabens and hormone levels or conventional semen quality parameters, but its positively associated with sperm DNA damage (83). Our retrospective study stated before revealed a significant decrease in sperm vitality and motility (progressive and non-progressive) in the exposed group. These results emphasize the negative impact of pesticides exposure on sperm DNA integrity. However, the sperm DNA integrity analysis includes the sperm DNA fragmentation demonstrating a significant increase of DNA fragmentation index in the group exposed to pesticides compared to the non-exposed group (86). Another study suggests that even smoking and alcohol consumption have detrimental effects on spermatogenesis through oxidative stress-induced changes. The findings show an increased catalase (CAT), superoxide dismutase (SOD) and glutathione reductase (GR) activities in the exposed group as well as the significant increase of DNA fragmentation. These increased enzymes activities suggest that the testicular cells are responding to elevated oxidative stress caused by smoking and alcohol that are environmental factors we are exposed to in addition to EDCs (138).

### 6.2. Epigenetic modification

#### 6.2.1. DNA methylation

**Figure 3:**
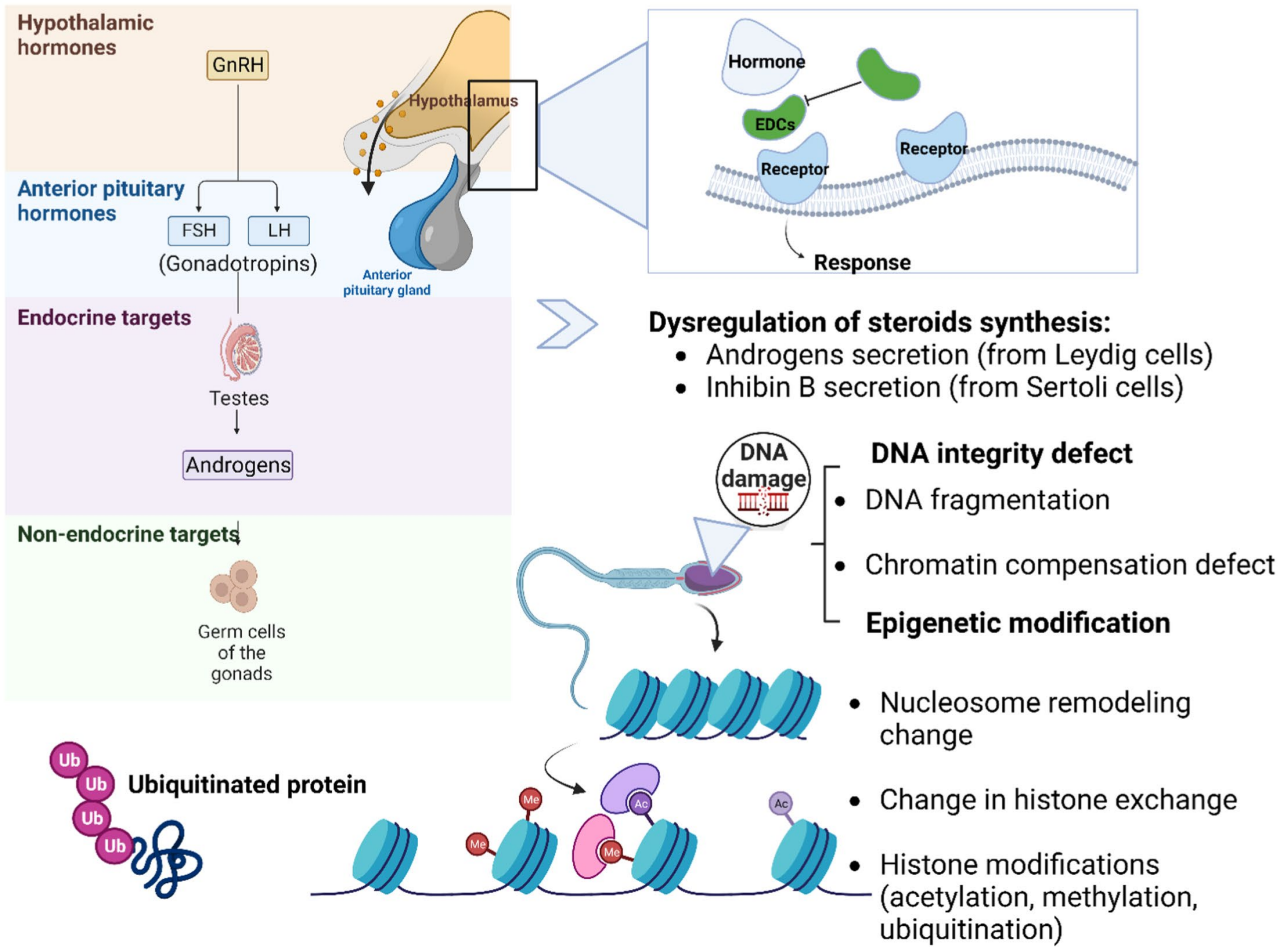
Schematic representation of possible mechanism actions of EDCs on DNA integrity and epigenetic changes. The EDCs (agonist or antagonist) disrupt the normal hormonal balance by mimicking, blocking, or altering the action of hormones in the body. These substances can bind to hormone receptors, interfere with hormone synthesis, metabolism, or transport, and disrupt the signaling pathways involved in hormone regulation such as the hypothalamo-pituitary-gonadal axis. Germ cells are the specialized cells in the gonads, responsible for transmitting genetic information from one generation to the next. The link between germ cells, epigenetic aspects, and DNA integrity is intertwined. DNA integrity is vital for the transmission of accurate genetic information. Various mechanisms work together to safeguard DNA integrity in germ cells. DNA repair pathways are active in germ cells to correct DNA damage and prevent the propagation of mutations to future generations. Epigenetic modifications (DNA methylation, histone modifications, and non-coding RNA molecules) is a heritable change in gene expression that does not involve alterations in the DNA sequence itself. This process helps establish and maintain the specific gene expression patterns required for proper germ cell differentiation and function. Additionally, the epigenetic landscape of germ cells can influence DNA integrity by regulating chromatin structure and accessibility, thereby protecting the genetic material from potential DNA damage.

It is true that DNA sequence damage or mutations can explain most pathologies. However, some abnormalities are associated with molecular factors that are not related to classic genetics or DNA mutations. Here we are referring to molecular factors/processes around the DNA that regulate genome activity that is independent of DNA sequence (139). These processes are mitotically stable and defined as epigenetics (140). In the literature, few studies have reported the negative impact of pesticides on epigenetic integrity. Systematic reviews of study reported that the impact of an environmental factor in altering epigenetic processes to promote gene expression and phenotypic changes, is defined as “environmental epigenetics” (9, 140, 141). In fact, EDCs may not have the ability to modify DNA sequence but can alter the DNA methylation patterns, induce histone modification and miRNA regulation leading to transcriptional changes associated with different diseases such as hormone production disruption (Figure 3), affecting neuroendocrine system dysfunction (142), prostate carcinogenesis process (95), change in behavior responses (73), and tumor formation in second generation (F2) mice (143, 144). Menezo et al. (145) reported that EDCs exert trans-generational effects on endocrine and metabolic function, as well as sperm epigenetic changes, including alterations in DNA methylation. DNA methylation is an important epigenetic modification that can affect gene expression, genomic imprinting, and genomic stability, and EDCs can impact DNA methylation patterns. They can also induce oxidative stress through their interactions with estrogen receptors (ER) and peroxisome proliferator-activated receptors (PPAR), which can lead to the damage of cellular components such as DNA, lipids, and proteins. This can have a range of negative health effects, including increased risk of chronic diseases such as cancer, diabetes, and cardiovascular disease (145).

There is increasing evidence that pregnancy status is associated with the development of reproductive tract cancers in both humans and animals. In 2003, Shuanfang and colleagues proposed that perinatal diethylstilbestrol (DES) exposure led to gene expression alteration and epigenetic methylation changes. To confirm this hypothesis, he evaluated the expression of the proto-oncogene c-*fos* in mice uterus neonatally exposed to DES (2 μg/pup/day on postnatal days 1–5). On one hand, they found that the uterine c-*fos* expression level was significantly 1.4 to 1.9 higher in the neonatal DES-exposed group compared to the non-exposed group. On the other hand, the results showed that the unmethylated cytosine–phosphate–guanine (CpGs) level in exon 4 was higher in neonatal DES-exposed group than that in control (143). In addition, experimental research of Anway and colleagues demonstrated that exposure of female rats, during gestation, to the fungicide vinclozolin, an antiandrogenic compound, promoted a transgenerational disease state in the F1 generation: a decrease in spermatogenic capacity linked to altered DNA methylation patterns in germ lines (146, 147). Later in 2012, Manikkam et al. analysis of DNA methylated regions (DMR) showed that dioxin (2,3,7,8-tetrachlorodibenzo[p]dioxin, TCDD) exposure of female rats from the 8th to the 14th day of gestation, corresponding to the development of gonads, increases the incidence of prostate and polycystic ovary diseases as well as ovarian primordial follicle loss in the F1 and F3 generations. They were able to identify 50 differentially DMR in gene promoters of the F3 generation sperm epigenome, linking dioxin gestating exposure to epigenetic transgenerational inheritance (148). Two years later, they similarly found that gestating methoxychlor exposure is associated with the increase of incidence of multiple diseases including kidney and ovary diseases in the F1 and F3 generations mainly transmitted through female germline (149). Parallelly, Skinner and colleagues demonstrated that DDT exposure led to kidney, prostate and ovary diseases and tumor development in adults of the F1 generation whereas in the F3 generation 50% of males and females developed obesity linked to a transgenerational transmission through female and male germlines, mainly DMR in genes associated with obesity. Similarly to pesticides that are considered EDCs, a study showed that the plastic hazard BPA may disrupt epigenetic regulation of *fggy* in mouse WATS, a carbohydrate kinase gene related to obesity, by shifting its promoter toward hypomethylation state leading to transcriptional suppression (150).

#### 6.2.2. Small non-coding RNAs

Non-coding RNAs, such as microRNAs (miRNAs) and long non-coding RNAs (lncRNAs), are molecules that play important roles in regulating gene expression (151, 152). Pesticide exposure may impact the expression and function of non-coding RNAs, leading to altered gene expression patterns. A review published in 2020 emphasized the significance of non-coding RNAs, particularly miRNAs and lncRNAs. It provided a comprehensive overview of how non-coding RNAs interact with environmental risk factors and their potential roles in disease development and response to environmental stressors. They highlight the complex interplay between genetic regulation, epigenetic modifications, and environmental influences (potentially aiding in assessing environmental risks). The study reported that lncRNAs may contribute to the response to environmental stressors. They could serve as exposure biomarkers, indicating whether an individual has been exposed to specific environmental stressors (153).

Giambo et al. (154) have reported that pesticides have the ability to influence the expression levels of various genes and trigger diverse epigenetic changes, affecting both miRNA expression levels and the regulation of DNA methylation status (154). Another study published in 2019 analyzed the alterations in rice transcriptomics subsequent to the application of two commercially available pesticides, Abamectin (ABM) and Thiamethoxam (TXM). The study outcome identified 1,140 differentially expressed genes (DEGs) that interact with 105 long non-coding RNAs (lncRNAs), potentially susceptible to be influenced by both pesticides (155).

#### 6.2.3. Histone modification

Epigenetic research, including the study of histone modifications, has gained significant attention in recent years due to its potential implications for various health outcomes, including those related to pesticide exposure (156). Numerous studies have investigated the effects of pesticides on histone modifications. These studies often utilize cell culture models, animal models, and sometimes human samples to assess changes in histone modifications following pesticide exposure (154).

Epigenetic alterations, including the implication in cancer, are now more clearly understood to have a critical role in both health and illness (157). Rafeeinia et al. investigated the association of organochlorine pesticides (OP) and the epigenetic changes including DNA methylation and histone modifications in children with lymphoblastic leukemia. The findings revealed that the OP are associated with significant increase in methylation at certain promoters and a decrease in the expression of certain histones including H4K16ac and H3K4me3 which may lead to leukemia in children (158). The Glyphosate exposure is associated with decrease in H3 acetylation and H3K27me3 methylation as well as increased H3K9 methylation and H4 acetylation in rats. Additionally, in mammalian stem cells, it is linked to small, single-stranded, non-coding RNA molecules. Glyphosate-induced epigenetic alterations can be transmitted to the progeny in the following generation (159).

Epigenetic modifications can sometimes be transferred from one generation to the next, a phenomenon known as transgenerational epigenetic inheritance. This means that changes in epigenetic marks caused by pesticide exposure in one generation could potentially influence the reproductive health and development of the offspring (9).

The potential of epigenetic modifications to mediate the effects of pesticide exposure on reproductive health highlights the complexity of environmental influences on human biology (156).

## 7. Discussion

In this review, we focused on endocrine disrupting chemicals because our current studies and research concern the effect of EDCs on male fertility. In a French study conducted within the Hauts-de-France region, known for its extensive agricultural activities and significant usage of pesticides identified as EDCs, Picardy, located in northern France, stands out for its substantial annual pesticide consumption. Approximately 3,900 tons of pesticides are utilized within the agricultural sector in this region (48).

Scientific research on the impact of EDCs on the reproductive system and male fertility has yielded a complex and sometimes conflicting body of evidence. It presents a multifaceted landscape with cross-talks and contrasting findings. While some studies have reported adverse effects, including altered hormone levels and reduced sperm quality, others have produced inconclusive results or failed to replicate these findings. A study by Meeker and Ferguson (160) have reported links between exposure to EDCs and adverse reproductive outcomes. These findings suggest potential impacts on hormone levels, sperm quality, and male fertility. However, Perheentupa (161) discusses both the supportive and contradictory evidence and the challenge in studies regarding the impact of EDCs on male reproductive health.

Contrary arguments and inconclusive results have also emerged in scientific literature. Some studies have failed to replicate the observed associations between EDCs exposure and male reproductive health. For example, a study by Boberg et al. (162) did not find significant adverse effects on male reproductive parameters (163). These conflicting findings can be attributed to variations in study design, exposure levels, and the complex interplay of genetic and environmental factors.

In a study conducted on greenhouse workers in Denmark, it was reported that there were no significant differences in sperm viability, sperm velocity, or levels of sex hormones between the pesticide-exposed group and the non-exposed group (164). Similarly, a Swedish conscripts study enrolled on 234 young men, did not show any significant associations between monobutyl or monobenzyl phthalate monoester and semen quality (165).

While these studies did not find significant differences in the parameters they examined, other Tunisian studies have shown different results. Ennaceur et al. (166) have reported that toxic pesticides including, dichlorodiphenytrichloroethane and its metabolites (DDTs), hexachlorobenzene (HCB), hexachlorocyclohexane isomers (HCHs), dieldrin, and polychlorinated biphenyls (PCBs) affects breastfeeding infants and maternal health (166, 167). Auriemma et al. demonstrated that environmental exposure to EDCs during fetal development could be contributing to the increase in male genital abnormalities, such as hypospadias, cryptorchidism and testicular germ-cell cancer (168). Developmental Origins of Health and Disease (DOHaD) studies suggested that critical windows of development is a sensitive period to environmental exposure during an early life (169, 170).

Going further, the relationship between pesticide exposure and non-Hodgkin lymphoma (NHL) remains an area of ongoing research. New studies and data may provide further insights into this potential association. Studies examining the association between pesticide exposure and Hodgkin lymphoma have produced mixed results. While some studies have suggested a potential link between pesticide exposure and an increased risk of NHL (171), others have not found a significant association (172).

Comparison between all these studies stated above proves that the impact of EDCs can vary depending on a plethora of factors, including the type and intensity of pesticide exposure, the duration and the period (perinatal or adulthood) of exposure, and individual variations in response and individual susceptibility. It is true that our study published in 2023 has reported that pesticide exposure did not appear to have a significant impact on certain sperm parameters, including volume, sperm count, sperm morphology and chromatin compaction (86). However, we were able to find a significant association between pesticide exposure and sperm DNA fragmentation. In fact, male infertility is a long-term consequence of exposure to contaminants. Thus, non-observed effects on the physiological level does not imply that EDCs have no effect on male fertility. Further analyses on the genetic and epigenetic level are necessary to decipher the link between exposure to EDCs and the emergence of infertility. Furthermore, researchers are currently focusing on the “exposome”: a concept used to describe environmental exposures that an individual encounters throughout life, and how these exposures impact biology and health (173). This concept suggests that while working on patients and analyzing the association between their infertility and their EDCs exposure, a screening of their life environmental exposures (of EDCs but also other xenobiotics) should be considered (blood, urine and feces samples for recent exposure and hair for old one) (174).

Unfortunately, one reason for the relative scarcity of reports on the effects of EDCs on humans is the difficulty in conducting controlled, long-term studies on human populations. Ethical concerns and practical limitations often restrict researchers to observational studies, which may not establish causation definitively. Additionally, the effects of these chemicals may manifest over a long period, making it challenging to link exposure to specific outcomes. The latency period for certain reproductive effects is often extended, making it difficult to link exposure to specific outcomes. Human exposure to EDCs is ubiquitous, making it challenging to establish clear cause-and-effect relationships. This ubiquitous exposure to EDCs in the environment contributes to difficulties in pinpointing individual exposures as causal factors in reproductive issues. Nonetheless, ongoing research continues to shed light on these issues, highlighting the need for further investigation and environmental regulations to minimize potential risks.

## 8. Conclusion

Endocrine disrupting chemicals have a harmful impact on human health, not only on exposed men but on the entire population. These chemicals, known as EDCs, are widespread and exist in various environmental domains. Human exposure occurs on a daily basis through sources like water, as well as through the consumption of food items such as vegetables and fruits, in addition to inhalation through the air. Their ability to bioaccumulate and their long half-life reflect their risk not only on plants and animals but also humans. In this present review, we summarized the reprotoxicity of some EDCs that might be associated with male infertility. Regulatory agencies like the Environmental Protection Agency (EPA) and the European Chemicals Agency (ECHA) conducted screening programs to assess the potential endocrine disrupting effects of various chemicals on human health and the environment. We discussed the mechanism of action in the hypothalamic–pituitary axis, the detrimental impact on testicular cells, spermatogenesis process and semen quality including total count, motility, and normal morphology. In addition to the damage of DNA integrity, we detailed their impact on epigenetic processes. These effects seem to be one of the reasons of hypofertility in men and it can explain a part of *in vitro* fertilization failures. The decline in male fertility has become a major public health issue, and exposure to EDCs is one of the factors that may contribute to this phenomenon. Therefore, it is important to minimize exposure to EDCs, both in the workplace and in the environment. In addition, more research is needed to better understand the mechanisms by which EDCs affect male reproductive function, and to develop effective strategies to prevent or treat the negative effects of EDCs on male fertility. However, to understand better the impact of EDCs, future prospective studies should consider assessing the impact of EDCs exposure histologically and immune-histologically of reproductive organs in rats. Finally, raising awareness about EDCs exposure in the population, especially the youngest categories, should be implemented to enhance the increase of life expectancy and slow the decline of male fertility.

## Author contributions

All authors listed have made a substantial, direct, and intellectual contribution to the work and approved it for publication.

## Figures and Tables

**Table:** 

EDCs	Endocrine disrupting chemicals
EDs	Endocrine disruptors
DNA	Deoxyribonucleic acid
GnRH	Gonadotropin-releasing hormone
FSH	Follicle-stimulating hormone
LH	Luteinizing hormone
ANSES	Agence Nationale de Sécurité Sanitaire de l’alimentation, de l’environnement et du travail
EPA	The Environmental Protection Agency
EFSA	The European Food Safety Authority
ECHA	The European Chemicals Agency
NRs	Nuclear receptors
PCB	Polychlorinated biphenyls
BPA	Bisphenol A
DBP	DiButyl Phthalate
DEHP	Di-(2-EthylHexyl) Phthalate
DDT	Dichloro-Diphenyl-Trichloroethane
CPF	Chlorpyrifos
HFD	High fat diet
PBDEs	Polybrominated diphenyl ethers
IVF	*In Vitro* fertilization
AR	Androgen receptor
TDI	Tolerable daily intake
SHBG	Sex hormone binding globulin
PFAs	PolyFluoroAlkyl substances
GBH	Glyphosate-based herbicides
ER	Endoplasmic reticulum
INSL3	Insulin-Like 3
ASD	Anoscrotal distance
AGD	Anogenital distance
APD	Anopenile distance
ER	Estrogen receptor
UPR	Unfolded protein response
PERK	Protein kinase R-like endoplasmic reticulum kinase
ATP	Adenosine triphosphate
ROS	Reactive oxygen species
CAT	Catalase
SOD	Superoxide dismutase
GR	Glutathione reductase
PPAR	Peroxisome proliferator-activated receptors
DES	DiEthylStilbestrol
CpGs	Cytosine–phosphate–Guanine
c-fos	Cellular-fos (fos = light in Greek) gene-encoded protein (a protooncogene)
DMR	DNA methylated regions
TCDD	2,3,7,8-TetraChloroDibenzo[p]Dioxin
miRNAs	Micro RiboNucleic acids
LncRNAs	Long non-coding RNAs
NcRNAs	Non-coding RNAs
ABM	Abamectin
TXM	Thiamethoxam
DEGs	Differentially expressed genes
eIF2α	Eukaryotic translation Initiation Factor 2α
ATF4	Activating transcription factor 4
AMPc	Cyclic adenosine monophosphate
P450scc	Side-chain cleavage enzyme
IKK	IkappaB kinase
AC	Adenylate cyclase
PKA	Protein kinase A
